# Effects of paprika carotenoid supplementation on bone turnover in postmenopausal women: a randomized, double-blind, placebo-controlled, parallel-group comparison study

**DOI:** 10.29219/fnr.v64.4565

**Published:** 2020-10-06

**Authors:** Naofumi Umigai, Yusuke Kozai, Tadahiro Saito, Tsuyoshi Takara

**Affiliations:** 1 Riken Vitamin Co., Ltd., Tokyo, Japan; 2 Department of Dentomaxillofacial Diagnosis and Treatment, Kanagawa Dental University, Kanagawa, Japan; 3 Medical Corporation Seishinkai, Takara Clinic, Tokyo, Japan

**Keywords:** osteoporosis, bone resorption, clinical trial, postmenopause, capsicum, capsanthin, β-carotene, β-cryptoxanthin, zeaxanthin

## Abstract

**Background:**

Paprika (*Capsicum annuum* L.) is a good source of carotenoids, including capsanthin, β-carotene, β-cryptoxanthin, and zeaxanthin. Several epidemiological studies have shown a beneficial association of intake of these carotenoids or their blood concentration with bone mineral density (BMD) and fracture risk. However, little information is available regarding the effect of intake of these carotenoids on bone metabolism in postmenopausal women.

**Objective:**

The objective of the present study was to investigate the effects of paprika carotenoid extract (PCE) on bone turnover in healthy, postmenopausal women.

**Design:**

We conducted a randomized, double-blind, placebo-controlled, parallel-group comparison study. One hundred participants were randomly assigned to PCE or placebo groups. Each group was given a 20 mg PCE (equivalent to 1.4 mg of carotenoids) a day or a placebo for 24 weeks. We measured bone resorption markers (tartrate-resistant acid phosphatase 5b [TRACP-5b] and serum type I collagen cross-linked N-telopeptide [sNTX]) at 12 and 24 weeks and bone formation markers (bone alkaline phosphatase and osteocalcin) at 24 weeks.

**Results:**

The percentage decrease of TRACP-5b at 24 weeks was significantly higher for PCE than the placebo. There were no significant differences in sNTX or bone formation markers, although PCE decreased each marker compared with the placebo.

**Conclusion:**

Our findings suggest that PCE supplementation suppresses bone resorption and contributes to maintaining bone quality in postmenopausal women.

## Popular scientific summary

- Paprika (*Capsicum annuum* L.) is a good source of carotenoids, including capsanthin, β-carotene, β-cryptoxanthin, and zeaxanthin.
- This clinical study evaluated the effects of paprika carotenoid extract (PCE) on bone turnover.
- Consecutive intake of PCE suppressed tartrate-resistant acid phosphatase 5b elevation in postmenopausal women.
- PCE supplementation may suppress bone resorption and contribute to maintaining bone quality in postmenopausal women.

Osteoporosis is a common skeletal disorder in the elderly population, characterized by a compromised bone strength and a predisposition to an increased risk of fracture (1). Osteoporosis mainly affects postmenopausal women due to the estrogen deficiency during the menopause transition, which induces high bone turnover with excessive bone resorption and rapid bone loss (2). In Japan, the prevalence of osteoporosis in women is three times higher than in men (men, 3 million; women, 9.8 million) (3, 4). Furthermore, with an expected increase of elderly populations, the number of patients with osteoporosis is also expected to increase. Osteoporotic fracture creates a significant financial burden and a markedly reduced health-related quality of life (5). Thus, osteoporosis is a growing concern for the aging population and is becoming a major public health problem.

Bone strength is determined not only by bone mineral density (BMD) but also by bone quality parameters such as bone microarchitecture, damage accumulation, mineralization, and bone turnover (1). Increases in BMD as a result of drug treatment do not necessarily correlate with reduced fracture risk (6). In addition, microarchitectural deterioration caused by high bone turnover remarkably increases bone fragility (7). Furthermore, bone loss in the early stages after menopause occurs mainly in the cancellous bone (8). The cancellous bone has a large surface exposed to the bone marrow and blood flow and thus exhibits higher turnover than the cortical bone (9). Bone turnover markers are believed to mainly reflect bone metabolism in cancellous bone, and high bone turnover reflected by elevated levels of bone turnover markers is believed to be a fracture predictor independent of BMD (4).

Nutritional approaches are essential in the development and maintenance of a healthy skeletal system. It is well known that calcium is an essential mineral for bone formation, and inadequate calcium intake during childhood and adolescence leads to insufficient bone mass (10). Vitamin D is a prohormone responsible for increasing the intestinal absorption of calcium and phosphate. Vitamin D deficiency leads to rickets in children and osteomalacia in adults (11). Calcium and vitamin D intake by the elderly may be beneficial in reducing the risk of osteoporotic fracture (12). In addition to these nutrients, phytochemicals found in fruits and vegetables have also been suggested to prevent osteoporosis.

Carotenoids are the pigments found in various fruits and vegetables, and some carotenoids are present in human blood as a result of consuming these foods (13). Several epidemiological studies have reported a beneficial association of carotenoid intake or its blood concentration with BMD and risk of fracture. For example, a higher intake of total carotenoids has been significantly associated with a lower hip fracture risk (14–16).

For individual carotenoids, the Mikkabi study found that serum concentrations of β-cryptoxanthin and β-carotene are inversely associated with the change of radial BMD in postmenopausal Japanese women (17). Similarly, a cross-sectional study conducted in Korea showed that the dietary intake of β-cryptoxanthin and β-carotene was significantly associated with a lower risk of osteopenia (18). On the other hand, a significant association between higher lycopene intake and a lower risk of hip or non-vertebral fracture was observed in the Framingham Osteoporosis Study (14). These epidemiological data suggest that adequate intake of carotenoids might be useful to maintain bone health. In addition, several *in vitro* and *in vivo* studies have shown that some carotenoids suppress bone resorption (19–22). However, few clinical studies have focused on the role of carotenoid supplementation in bone turnover. A previous study reported that β-cryptoxanthin administration in ovariectomized mice contributed to trabecular structure improvement rather than BMD (23). Daily carotenoid supplementation is expected to suppress the high bone turnover and maintain bone quality in postmenopausal women.

The fruits of *Capsicum annuum* L. are generally referred to as paprika or pepper and contain a variety of carotenoids. Capsanthin, a typical pigment in the capsicum species, is the most abundant carotenoid, but other carotenoids, such as β-carotene, β-cryptoxanthin, and zeaxanthin, are also present (24). Carotenoids extracted from paprika have been widely used as natural coloring agents for various foods such as meat products, confectioneries, soups, and sauces. In recent years, there has been an increased interest in the pharmacological activities of paprika carotenoids as well as their role as an antioxidant (25), anti-inflammatory (26), and anti-obesity (27) agent.

In the present study, we investigated the effects of paprika carotenoid extract (PCE) on bone metabolism in healthy postmenopausal women. This study may provide further insights into a nutritional approach for bone health maintenance.

## Materials and methods

### Study design

We initiated a randomized, double-blind, placebo-controlled, parallel-group comparison study, including a screening period of 7 weeks and an intake period of 24 weeks. This study was performed at Takara Clinic (Tokyo, Japan) from July 2018 to March 2019. The study was approved by the Medical Corporation Seishinkai Takara Clinic Ethics Committee (approval no. 1806-1805-RV02-01-TC), and the protocol was registered with the University Hospital Medical Information Network Clinical Trials Registry (UMIN000033180). This study was conducted following the principles of the Declaration of Helsinki and the Ethical Guidelines for Medical and Health Research Involving Human Subjects issued by the Japanese Ministry of Education, Culture, Sports, Science and Technology and Ministry of Health, Labour and Welfare. All participants provided a written informed consent before any study procedures were initiated.

### Participants

The participants were recruited from an online website operated by ORTHOMEDICO Inc. (Tokyo, Japan). The inclusion criteria were as follows: healthy Japanese women aged over 40 years and amenorrhea over 12 months, which we considered as natural postmenopause. The exclusion criteria were as follows: diagnosed with osteoporosis (less than 70% in total amount of BMD for the lumbar vertebra of the young adult mean score); possible allergy to the raw materials of the test supplements; the use of medication or supplements for bone metabolism; a smoking habit; a history of major diseases such as heart, liver, kidney, cerebrovascular, or digestive system diseases; a history of rheumatism, diabetes mellitus, or hypertension; and anyone judged unsuitable by the physician in charge. The participants were continuously recruited during the screening period from July 6 to August 25, 2018.

The participants were required to visit the clinic for screening, where their health condition and eligibility were assessed. The participants were fully informed about the test supplements and study methods before providing written informed consent.

### Test supplements

**Fig. 1:**
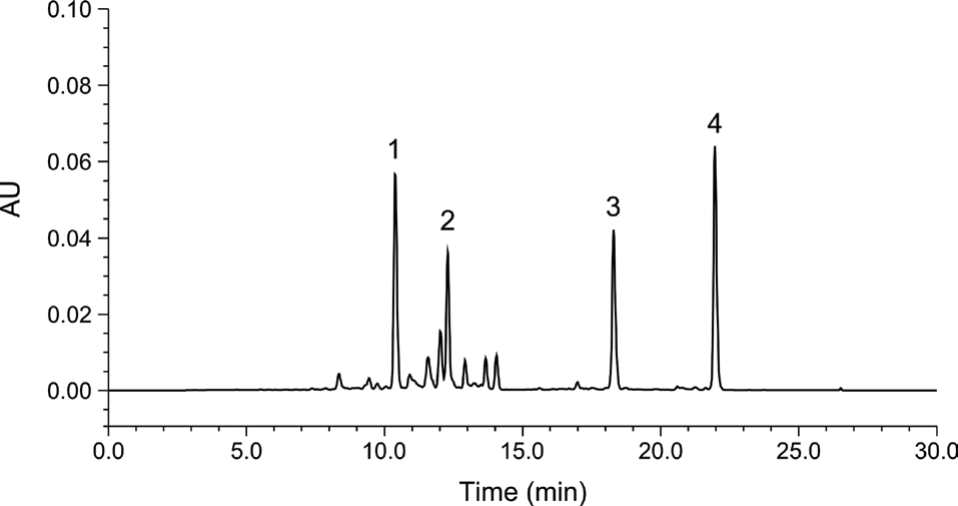
Representative chromatogram after the saponification of the paprika carotenoid extract used in this study. Peak identification: 1, capsanthin; 2, zeaxanthin; 3, β-cryptoxanthin; 4, β-carotene.

A commercial PCE with a total standardized content (≥7%) of the four major carotenoids, namely, capsanthin, β-carotene, β-cryptoxanthin, and zeaxanthin, was purchased from Riken Vitamin (Tokyo, Japan). This carotenoid extract was analyzed for its carotenoid profile using reversed-phase high-performance liquid chromatography (HPLC) methods as follows: the HPLC system used was a Waters e2695 Separation Module (Waters, Milford, MA, USA). The stationary phase used was TSKgel ODS-80Ts columns (5 μm, 4.6 mm ID × 250 mm; Tosoh, Japan) at 35°C. The mobile phases comprised solvent A (water) and solvent B (acetone) delivered at a flow rate of 1.0 mL/min and analyte was detected at 450nm. Gradient elution was started with 72% B and held for 2 min, reaching 90% B at 12 min, 96% B at 16 min, and ultimately, 100% B at 20 min. From 23 to 35 min, the system was re-equilibrated with the initial composition (28% A and 72% B). The PCE used in this study was found to contain the following carotenoids: 2.78% capsanthin, 2.13% β-carotene, 1.54% β-cryptoxanthin, and 1.21% zeaxanthin (Fig. 1).

The test supplements were prepared as soft capsules. The test capsules were filled with 140 mg of rapeseed oil, 40 mg of emulsifier, and 20 mg of PCE, with each capsule containing 1.4 mg of carotenoids. The placebo capsules were identical to the test capsules apart from replacing PCE with rapeseed oil. The nutritional composition of the test supplements is shown in Table 1. The test and placebo capsules were indistinguishable in size, taste, flavor, and appearance. Each capsule was packaged in identical aluminum bags labeled with an identification number.

**Table 1:** Nutritional composition of the test supplements (per one capsule)

Composition	Test supplement (330 mg)	Placebo (330 mg)
Energy, kcal	2.21	2.22
Protein, g	0.102	0.102
Fat, g	0.186	0.186
Carbohydrate, g	0.033	0.033
Sodium, mg	0.006	0.005
Paprika carotenoid extract (PCE), mg	20	–
Carotenoids^†^, mg	1.4	–

† Total content of the carotenoids: capsanthin, β-carotene, β-cryptoxanthin, and zeaxanthin.

### Randomization

Randomization was stratified by bone resorption marker values and years after menopause at the screening. The randomization list was generated by dedicated staff at an independent third-party organization using the software StatLight (Yukms, Tokyo, Japan), which allocated the participants to two groups. The allocation information was blinded to the investigator, clinical staff, participants, and anyone else directly involved in conducting the study until the database was locked.

### Procedure

The participants visited the clinic on three occasions: at screening, including the baseline assessment (visit 1), then at 12 and 24 weeks (visits 2 and 3) for the intake period. Bone resorption markers (tartrate-resistant acid phosphatase 5b [TRACP-5b] and serum type I collagen cross-linked N-telopeptide [sNTX]) were measured at every visit. Bone formation markers (bone alkaline phosphatase [BAP] and osteocalcin [OC]) were measured at visits 1 and 3. At each visit, the participants underwent a physical examination, blood test, biochemical blood test, urine analysis, and medical interview to evaluate safety. During the intake period, each participant took one capsule daily with water after breakfast. The participants began intake sequentially from September 10, 2018 and were followed up with until March 15, 2019.

During the intake period, the participants were instructed to take the test supplements daily by not changing their lifestyle habits such as diet, drinking, and exercise. In addition, the participants were asked, as far as possible, to avoid intake of carotenoid-rich foods, such as vegetable or fruit juices. The participants recorded their daily steps using a pedometer and were asked to record their activities and health condition in a daily diary. Adherence was monitored by capsule count (number dispensed and returned) and by the recorded daily diary and steps.

### Outcomes

The primary efficacy endpoint was defined as the percentage change in bone resorption markers from baseline to the end of the test supplement intake period (24 weeks). The secondary efficacy endpoint was the percentage change in bone formation markers.

### Safety evaluations

Safety evaluations comprised physical examinations of body weight, body mass index, systolic pressure, diastolic pressure, pulse rate, and body temperature. Hematological analyses were a collection of white blood cell counts, red blood cell counts, hemoglobin, hematocrit, mean corpuscular volumes, mean corpuscular hemoglobin, mean corpuscular hemoglobin concentrations, platelet counts, neutrophils, lymphocytes, monocytes, eosinophils, and basophils. The measurement of the blood biochemical parameters included aspartate aminotransferase, alanine aminotransferase, γ-glutamyl transpeptidase, alkaline phosphatase, lactate dehydrogenase, leucine aminopeptidase, total-bilirubin, direct-bilirubin, indirect-bilirubin, cholinesterase, total protein, blood urea nitrogen, creatinine, uric acid, creatinine kinase, sodium, potassium, chlorine, calcium, inorganic phosphorus, serum iron, amylase, total-cholesterol, low-density lipoprotein cholesterol, high-density lipoprotein cholesterol, triglyceride, glucose, glycoalbumin, and hemoglobin A1c. The urinalysis tests for protein, glucose, urobilinogen, bilirubin, pH, uric blood, and urinary ketone.

### Statistical analysis

The participant characteristics are presented as mean ± standard deviation. The percentage change in bone turnover markers is expressed as mean ± standard error. Differences in participant characteristics and efficacy outcomes between the PCE and placebo intake groups were analyzed using the Student’s *t*-test, and the significance was set at *P* < 0.05. The software used for analyses was SPSS version 22.0 (IBM Corporation, Armonk, NY, USA).

We calculated the sample size via power analysis using G* power program (https://www.psychologie.hhu.de/arbeitsgruppen/allgemeine-psychologie-und-arbeitspsychologie/gpower.html) (28). Assuming an α of 0.05 (Student’s *t*-test, two sided) and a power of 80% to detect a medium effect size (0.60), 45 participants were required in each group. A dropout rate of 10% was considered, resulting in 50 participants per group.

## Results

### Characteristics of the participants

**Fig. 2:**
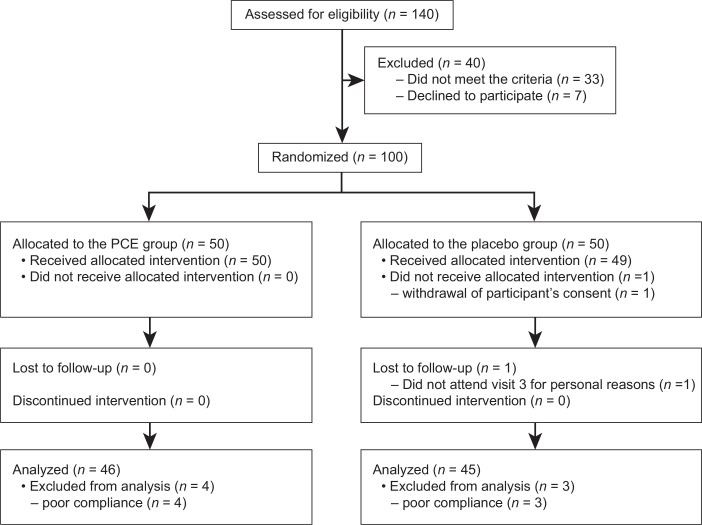
The flow of participants throughout the study.

The flow of participants through the study is shown in Fig. 2. A total of 140 potential participants were initially screened, and 100 were enrolled in the study. One participant in the placebo group did not receive the allocated intervention due to consent withdrawal, and one participant in the placebo group did not complete all of the clinical visits for personal reasons. Four participants in the PCE group and three participants in the placebo group were eliminated from the analysis due to poor compliance (i.e. frequently forgetting to take the test supplements and writing in the daily diary). The characteristics of the participants in the per-protocol analysis are shown in Table 2.

**Table 2:** Baseline characteristics of the participants in the per-protocol analysis

Characteristics	Paprika carotenoid extract (PCE) group (*n* = 46)	Placebo group (*n* = 45)	*P*-value
Age, years	56.3 ± 6.1	56.9 ± 5.8	0.628
Height, cm	157.4 ± 4.5	157.0 ± 4.7	0.672
Weight, kg	51.6 ± 4.8	53.4 ± 8.8	0.240
Body mass index (BMI)	20.9 ± 2.0	21.7 ± 3.5	0.181
Time since menopause, years	6.8 ± 5.7	6.6 ± 5.7	0.857
Bone resorption markers			
Tartrate-resistant acid phosphatase 5b (TRACP-5b), mU/dL	409.8 ± 141.8	411.4 ± 154.4	0.959
Serum type I collagen cross-linked N-telopeptide (sNTX), nmol BCE/L	18.5 ± 5.0	18.4 ± 4.4	0.934
Bone formation markers			
Bone alkaline phosphatase (BAP), μg/L	13.4 ± 3.7	12.4 ± 3.5	0.202
Osteocalcin (OC), ng/mL	17.1 ± 5.8	18.0 ± 7.1	0.509

Values are expressed as mean ± standard deviation.

### Percentage change from baseline in bone turnover markers

**Fig. 3:**
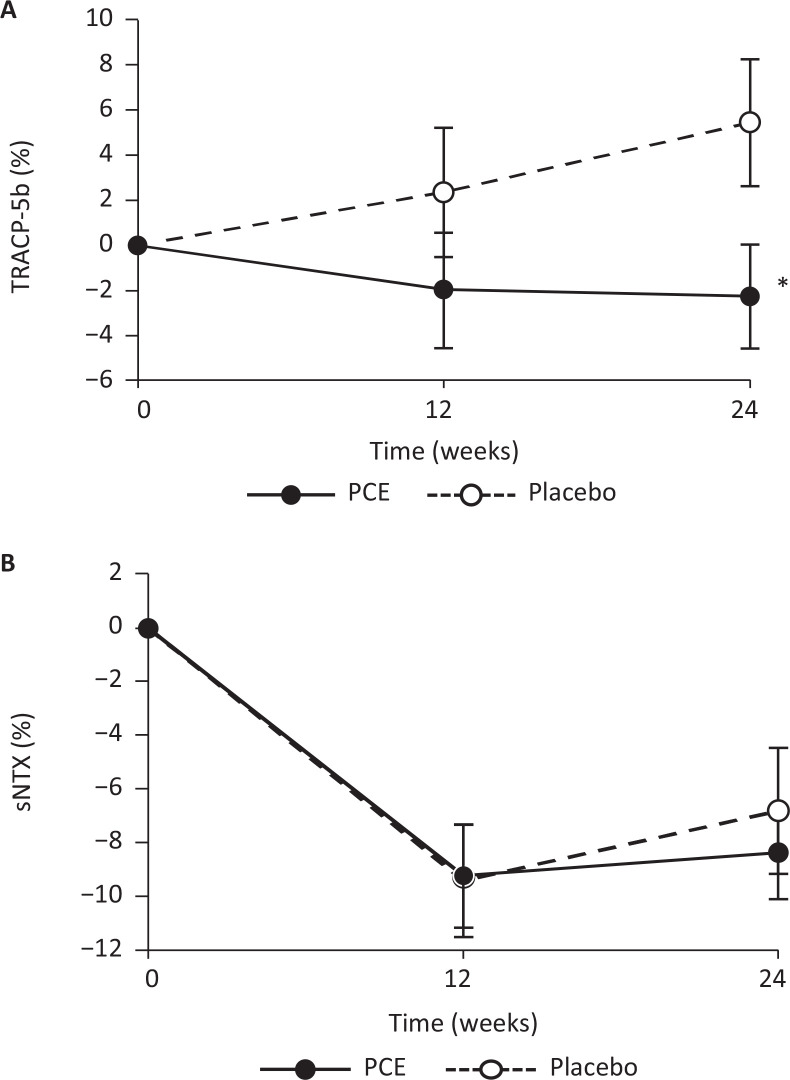
Percentage change from baseline to 24 weeks of intake in (a) tartrate-resistant acid phosphatase 5b (TRACP-5b) and (b) serum type I collagen cross-linked N-telopeptide (sNTX). Values are presented as mean ± standard error. Asterisks represent *P* < 0.05 compared between the groups.

The TRACP-5b in the PCE group gradually declined, whereas in the placebo group, there was a rise (Fig. 3a). At 24 weeks, the percentage change was −2.3 ± 2.3% with the PCE and 5.5 ± 2.8% with the placebo. There was a significant difference between the groups (mean difference, −7.7; 95% confidence interval, −15.0 to −0.5; *P* = 0.037). There was no observed significant difference in sNTX (Fig. 3b) between the groups at 24 weeks (PCE group, −8.4 ± 1.8%; placebo group, −6.8 ± 2.4%; mean difference, −1.6; 95% confidence interval, −7.4 to 4.3; *P* = 0.595). No significant differences between the groups were found for the percentage change from the baseline in bone formation marker levels, although PCE decreased each marker’s level compared with the placebo (Table 3).

**Table 3:** Percentage change in bone formation markers from baseline to 24 weeks of intake

Variables	Paprika carotenoid extract (PCE) group (*n* = 46)	Placebo group (*n* = 45)	Between group difference mean (95% confidence interval [CI])	*P*-value
Bone alkaline phosphatase (BAP), %	9.1 ± 2.7	12.2 ± 2.3	−3.1 (−10.1 to 4.0)	0.386
Osteocalcin (OC), %	−6.6 ± 2.5	−4.6 ± 2.6	−1.9 (−9.2 to 5.3)	0.599

Values are expressed as mean ± standard error.

### Safety evaluations and adverse events

During the physical examination, blood test, biochemical blood test, and urine analysis, several significant changes were sporadically observed. However, these changes were small and within the normal range (data not shown). Adverse events were reported during the intake period, including 25 cases of cold-like symptoms (11 in the PCE group, 14 in the placebo group), 10 cases of headaches (eight in the PCE group, two in the placebo group), eight cases of influenza (six in the PCE group, two in the placebo group), four cases of pollen allergy (two in the PCE group, two in the placebo group), five cases of joint pain (one in the PCE group, four in the placebo group), two cases of gastralgia (one in the PCE group, one in the placebo), one case of urticaria in the PCE group, and one case each of gastroenteritis, constipation, and cough variant asthma in the placebo group. Following clinical interviews and safety evaluations, all of these adverse events were evaluated by the study physician to be unrelated to the test supplements.

## Discussion

In the present study, we observed that oral PCE supplementation suppressed TRACP-5b elevation in postmenopausal women. Meanwhile, no significant change in bone formation markers was observed. In the safety evaluation, no adverse effect was observed related to the PCE. These results suggest that PCE supplementation contributes to moderate the excessive bone resorption outpacing bone formation after menopause.

Bone is a metabolically active tissue that undergoes constant remodeling throughout life. Bone integrity is maintained by bone remodeling, consisting of bone resorption by osteoclasts and bone formation by osteoblasts (29). The negative imbalance of bone turnover after menopause, which is excessive bone resorption outpacing the rate of bone formation, leads to a lower bone mass and microarchitectural deterioration (8). Thus, balancing bone resorption and bone formation is important for maintaining bone strength. Bone turnover markers are the useful indicators to assess bone metabolism because they can accurately express the status of daily bone turnover and are easily measured (30). Growing evidence shows the possibility of bone turnover markers as predictors for bone loss (31–33) and osteoporotic fracture risk (34–36).

In this study, TRACP-5b in the placebo group continued to increase during the intake period. It has been reported that urinary NTX, which is a bone resorption marker, begins increasing 2 years before the final menstrual period (FMP). It reaches a peak approximately 1.5 years after the FMP, then declines slightly and maintains higher levels than before the menopause transition (37). As the participants of the present study included women who were only a few years after menopause, a continuous increase of TRACP-5b may have been observed. However, PCE suppressed TRACP-5b elevation, and the percentage decrease from baseline to 24 weeks was significantly higher than with the placebo. TRACP-5b is an enzyme secreted by osteoclasts; its activity correlates with the number of osteoclasts and reflects the state of bone resorption (38).

Capsanthin, β-carotene, β-cryptoxanthin, and zeaxanthin are major carotenoids present in the PCE used in this study. In agreement with our findings, several studies have reported that these carotenoids inhibit osteoclastogenesis (19–21). Therefore, PCE may suppress bone resorption by decreasing the number of osteoclasts. In addition, it has been reported that TRACP-5b is predictive for fractures associated with osteoporosis (36). Epidemiological studies have reported that a higher intake of some carotenoids, including β-cryptoxanthin, β-carotene, and zeaxanthin, was favorably associated with a lower risk of osteoporotic fracture (15, 16). The present results partially support these epidemiological findings.

Meanwhile, sNTX was decreased in both groups, and no significant difference between the groups was observed. However, the percentage decrease at 24 weeks in the PCE group was slightly higher than in the placebo group. The reason that PCE had a limited effect on sNTX is unclear. NTX is a breakdown product of type I collagen in bone. This marker is affected by renal impairment and diurnal variations in sNTX more than in TRACP-5b (26). Additionally, it has been reported that TRACP-5b might be more sensitive to changes in bone resorption than sNTX (39). The antiresorptive effect of PCE in this study was mild. Therefore, a significant difference in sNTX was not observed.

The process of bone resorption and formation is tightly coupled, and bone formation follows bone resorption (29). Bone resorption markers decrease earlier than bone formation markers with the treatment of bone antiresorptive drugs, such as bisphosphonate (40). In the present study, although a significant decrease in TRACP-5b was observed, no significant differences between the groups were observed for bone formation markers 24 weeks after intake. The treatment duration in this study may have been insufficient to evaluate changes in bone formation.

In the safety evaluation, no adverse events were observed to be related to the PCE intake during the study period, indicating that PCE can be used safely at the dosage administered in this study. This study has several limitations that need to be considered. First, the plasma carotenoid concentration of the participants was not measured and, therefore, the characteristics are unknown. The effect of PCE on bone turnover observed in this study was mild. This may have been affected by the initial plasma carotenoid levels. We recommend that studies restrict participants to those who have low blood levels of carotenoids to avoid the influence of the initial carotenoid levels.

Furthermore, changes in BMD were not evaluated because the participants in this study were healthy postmenopausal women with high BMD values. Epidemiological studies indicate that high carotenoid intake and high blood carotenoid concentrations contribute to the maintenance of BMD (17, 18). The suppression of bone resorption by PCE intake observed in this study may prevent BMD reduction. A further investigation on whether PCE administration improves BMD is required.

Additionally, although both women and men are affected by osteoporosis, only postmenopausal women were included in this study. Thus, the results may not be generalizable to the whole adult population. However, the mechanism of PCE on the suppression of bone resorption would be different from the estrogen replacement effect, and epidemiological studies have reported a beneficial association between carotenoid intake and fracture risk in men (15). Suppression of bone resorption by PCE supplementation might be promising in men as well as women. Further studies are required to elucidate the efficacy of PCE in men.

## Conclusion

This study showed the effects of PCE on bone turnover in healthy postmenopausal women, including a decrease in TRACP-5b. PCE supplementation may suppress bone resorption, which contributes to the maintenance of bone quality in postmenopausal women.

## References

[1] NIH Consensus Development Panel on Osteoporosis Prevention, Diagnosis, and Therapy Osteoporosis prevention, diagnosis, and therapy. JAMA 2001; 285(6): 785–95. doi: 10.1001/jama.285.6.785 11176917

[2] Karlamangla AS, Burnett-Bowie SM, Crandall CJ Bone health during the menopause transition and beyond. Obstet Gynecol Clin North Am 2018; 45(4): 695–708. doi: 10.1016/j.ogc.2018.07.012 30401551 PMC6226267

[3] Yoshimura N, Muraki S, Oka H, Mabuchi A, En-Yo Y, Yoshida M, et al Prevalence of knee osteoarthritis, lumbar spondylosis, and osteoporosis in Japanese men and women: the research on osteoarthritis/osteoporosis against disability study. J Bone Miner Metab 2009; 27: 620–8. doi: 10.1007/s00774-009-0080-8 19568689

[4] Orimo H, Nakamura T, Hosoi T, Iki M, Uenishi K, Endo N, et al Japanese 2011 guidelines for prevention and treatment of osteoporosis – executive summary. Arch Osteoporos 2012; 7: 3–20. doi: 10.1007/s11657-012-0109-9 23203733 PMC3517709

[5] Fujiwara S, Zhao X, Teoh C, Jaffe DH, Taguchi Y Disease burden of fractures among patients with osteoporosis in Japan: health-related quality of life, work productivity and activity impairment, healthcare resource utilization, and economic costs. J Bone Miner Metab 2019; 37: 307–18. doi: 10.1007/s00774-018-0916-1 29520508

[6] Watts NB, Geusens P, Barton IP, Felsenberg D Relationship between changes in BMD and nonvertebral fracture incidence associated with risedronate: reduction in risk of nonvertebral fracture is not related to change in BMD. J Bone Miner Res 2005; 20(12): 2097–104. doi: 10.1359/JBMR.050814 16294263

[7] Zaidi M, Turner CH, Canalis E, Pacifici R, Sun L, Iqbal J, et al Bone loss or lost bone: rationale and recommendations for the diagnosis and treatment of early postmenopausal bone loss. Curr Osteoporos Rep 2009; 7: 118–26. doi: 10.1007/s11914-009-0021-4 19968915

[8] Eastell R, O’Neill TW, Hofbauer LC, Langdahl B, Reid IR, Gold DT, et al Postmenopausal osteoporosis. Nat Rev Dis Primers 2016; 2: 16069. doi: 10.1038/nrdp.2016.69 27681935

[9] Ott SM Cortical or trabecular bone: what’s the difference? Am J Nephrol 2018; 47: 373–5. doi: 10.1159/000489672 29788030

[10] Matkovic V, Fontana D, Tominac C, Goel P, Chesnut CH, 3rd. Factors that influence peak bone mass formation: a study of calcium balance and the inheritance of bone mass in adolescent females. Am J Clin Nutr 1990; 52(5): 878–88. doi: 10.1093/ajcn/52.5.878 2239765

[11] Christodoulou S, Goula T, Ververidis A, Drosos G Vitamin D and bone disease. Biomed Res Int 2013; 2013: 396541. doi: 10.1155/2013/396541 PMC3591184 23509720

[12] Prentice A Diet, nutrition and the prevention of osteoporosis. Public Health Nutr 2004; 7(1a): 227–43. doi: 10.1079/PHN2003590 14972062

[13] Eggersdorfer M, Wyss A Carotenoids in human nutrition and health. Arch Biochem Biophys 2018; 652: 18–26. doi: 10.1016/j.abb.2018.06.001 29885291

[14] Sahni S, Hannan MT, Blumberg J, Cupples LA, Kiel DP, Tucker KL Protective effect of total carotenoid and lycopene intake on the risk of hip fracture: a 17-year follow-up from the Framingham Osteoporosis Study. J Bone Miner Res 2009; 24(6): 1086–94. doi: 10.1359/jbmr.090102 19138129 PMC2683648

[15] Dai Z, Wang R, Ang LW, Low YL, Yuan JM, Koh WP Protective effects of dietary carotenoids on risk of hip fracture in men: the Singapore Chinese Health Study. J Bone Miner Res 2014; 29(2): 408–17. doi: 10.1002/jbmr.2041 23857780 PMC3894263

[16] Cao WT, Zeng FF, Li BL, Lin JS, Liang YY, Chen YM Higher dietary carotenoid intake associated with lower risk of hip fracture in middle-aged and elderly Chinese: a matched case-control study. Bone 2018; 111: 116–22. doi: 10.1016/j.bone.2018.03.023 29605302

[17] Sugiura M, Nakamura M, Ogawa K, Ikoma Y, Yano M High serum carotenoids associated with lower risk for bone loss and osteoporosis in post-menopausal Japanese female subjects: prospective cohort study. PLoS One 2012; 7: e52643. doi: 10.1371/journal.pone.0052643 23285126 PMC3527562

[18] Regu GM, Kim H, Kim YJ, Paek JE, Lee G, Chang N, et al Association between dietary carotenoid intake and bone mineral density in Korean adults aged 30–75 years using data from the Fourth and Fifth Korean National Health and Nutrition Examination Surveys (2008–2011). Nutrients 2017; 9(9): 1025. doi: 10.3390/nu9091025 PMC5622785 28926945

[19] Ozaki K, Okamoto M, Fukasawa K, Iezaki T, Onishi Y, Yoneda Y, et al Daily intake of β-cryptoxanthin prevents bone loss by preferential disturbance of osteoclastic activation in ovariectomized mice. J Pharmacol Sci 2015; 129(1): 72–7. doi: 10.1016/j.jphs.2015.08.003 26342276

[20] Hirata N, Ichimaru R, Tominari T, Matsumoto C, Watanabe K, Taniguchi K, et al Beta-cryptoxanthin inhibits lipopolysaccharide-induced osteoclast differentiation and bone resorption via the suppression of inhibitor of NF-κB kinase activity. Nutrients 2019; 11(2): 368. doi: 10.3390/nu11020368 PMC6412436 30744180

[21] Wang F, Wang N, Gao Y, Zhou Z, Liu W, Pan C, et al β-Carotene suppresses osteoclastogenesis and bone resorption b-y suppressing NF-κB signaling pathway. Life Sci 2017; 174: 15–20. doi: 10.1016/j.lfs.2017.03.002 28263804

[22] Rao LG, Krishnadev N, Banasikowska K, Rao AV Lycopene I-effect on osteoclasts: lycopene inhibits basal and parathyroid hormone-stimulated osteoclast formation and mineral resorption mediated by reactive oxygen species in rat bone marrow cultures. J Med Food 2003; 6(2): 69–78. doi: 10.1089/109662003322233459 12935316

[23] Iino M, Kozai Y, Kawamata R, Wakao H, Sakurai T, Kashima I Effects of β-cryptoxanthin on bone-formation parameters in the distal femoral epiphysis of ovariectomized mice. Oral Radiol 2014; 30: 1–8. doi: 10.1007/s11282-013-0131-7

[24] Rodriguez-Uribe L, Guzman I, Rajapakse W, Richins RD, O’Connell MA. Carotenoid accumulation in orange-pigmented Capsicum annuum fruit, regulated at multiple levels. J Exp Bot 2012; 63(1): 517–26. doi: 10.1093/jxb/err302 21948863 PMC3245482

[25] Nishino A, Yasui H, Maoka T Reaction of paprika carotenoids, capsanthin and capsorubin, with reactive oxygen species. J Agric Food Chem 2016; 64(23): 4786–92. doi: 10.1021/acs.jafc.6b01706 27229653

[26] Hernández-Ortega M, Ortiz-Moreno A, Hernández-Navarro MD, Chamorro-Cevallos G, Dorantes-Alvarez L, Necoechea-Mondragón H Antioxidant, antinociceptive, and anti-inflammatory effects of carotenoids extracted from dried pepper (*Capsicum annuum* L.). J Biomed Biotechnol 2012; 2012: 524019. doi: 10.1155/2012/524019 PMC3468166 23091348

[27] Kakutani R, Hokari S, Nishino A, Ichihara T, Sugimoto K, Takaha T, et al Effect of oral paprika xanthophyll intake on abdominal fat in healthy overweight humans: a randomized, double-blind, placebo-controlled study. J Oleo Sci 2018; 67(9): 1149–62. doi: 10.5650/jos.ess18076 30111683

[28] Faul F, Erdfelder E, Lang AG, Buchner A G*Power 3: a flexible statistical power analysis program for the social, behavioral, and biomedical sciences. Behav Res Methods 2007; 39: 175–91. doi: 10.3758/BF03193146 17695343

[29] Hadjidakis DJ, Androulakis II Bone remodeling. Ann N Y Acad Sci 2006; 1092(1): 385–96. doi: 10.1196/annals.1365.035 17308163

[30] Nishizawa Y, Miura M, Ichimura S, Inaba M, Imanishi Y, Shiraki M, et al Executive summary of the Japan Osteoporosis Society Guide for the Use of Bone Turnover Markers in the Diagnosis and Treatment of Osteoporosis (2018 Edition). Clin Chim Acta 2019; 498: 101–7. doi: 10.1016/j.cca.2019.08.012 31425674

[31] Iki M, Morita A, Ikeda Y, Sato Y, Akiba T, Matsumoto T, et al Biochemical markers of bone turnover predict bone loss in perimenopausal women but not in postmenopausal women-the Japanese Population-based Osteoporosis (JPOS) Cohort Study. Osteoporos Int 2006; 17: 1086–95. doi: 10.1007/s00198-005-0052-3 16758145

[32] Yoshimura N, Muraki S, Oka H, Kawaguchi H, Nakamura K, Akune T Biochemical markers of bone turnover as predictors of osteoporosis and osteoporotic fractures in men and women: 10-year follow-up of the Taiji cohort. Mod Rheumatol 2011; 21(6): 608–20. doi: 10.1007/s10165-011-0455-2 21512822

[33] Shieh A, Ishii S, Greendale GA, Cauley JA, Lo JC, Karlamangla AS Urinary N-telopeptide and rate of bone loss over the menopause transition and early postmenopause. J Bone Miner Res 2016; 31(11): 2057–64. doi: 10.1002/jbmr.2889 27322414 PMC5407063

[34] Garnero P, Hausherr E, Chapuy MC, Marcelli C, Grandjean H, Muller C, et al Markers of bone resorption predict hip fracture in elderly women: the EPIDOS prospective study. J Bone Miner Res 1996; 11(10): 1531–8. doi: 10.1002/jbmr.5650111021 8889854

[35] Ross PD, Kress BC, Parson RE, Wasnich RD, Armour KA, Mizrahi IA Serum bone alkaline phosphatase and calcaneus bone density predict fractures: a prospective study. Osteoporos Int 2000; 11: 76–82. doi: 10.1007/s001980050009 10663362

[36] Gerdhem P, Ivaska KK, Alatalo SL, Halleen JM, Hellman J, Isaksson A, et al Biochemical markers of bone metabolism and prediction of fracture in elderly women. J Bone Miner Res 2004; 19(3): 386–93. doi: 10.1359/JBMR.0301244 15040826

[37] Sowers MR, Zheng H, Greendale GA, Neer RM, Cauley JA, Ellis J, et al Changes in bone resorption across the menopause transition: effects of reproductive hormones, body size, and ethnicity. J Clin Endocrinol Metab 2013; 98(7): 2854–63. doi: 10.1210/jc.2012-4113 23666961 PMC3701268

[38] Lv Y, Wang G, Xu W, Tao P, Lv X, Wang Y Tartrate-resistant acid phosphatase 5b is a marker of osteoclast number and volume in RAW 264.7 cells treated with receptor-activated nuclear κB ligand. Exp Ther Med 2015; 9(1): 143–6. doi: 10.3892/etm.2014.2071 PMC4247282 25452790

[39] Tsuji O, Urakado M, Koyanagi E, Shinohara M, Hisae M, Naruo S, et al Comparison between TRACP-5b and serum NTX after treatment with risedronate in osteoporosis. Orthop & Traumatol 2011; 60(3): 473–6 (in Japanese). doi: 10.5035/nishiseisai.60.473

[40] Vasikaran S, Eastell R, Bruyère O, Foldes AJ, Garnero P, Griesmacher A, et al Markers of bone turnover for the prediction of fracture risk and monitoring of osteoporosis treatment: a need for international reference standards. Osteoporos Int 2011; 22: 391–420. doi: 10.1007/s00198-010-1501-1 21184054

